# A comparative mRNA- and miRNA transcriptomics reveals novel molecular signatures associated with metastatic prostate cancers

**DOI:** 10.3389/fgene.2022.1066118

**Published:** 2022-11-16

**Authors:** Thoraia Shinawi, Khalidah Khalid Nasser, Fatima Amanullah Moradi, Abdulrahman Mujalli, Walaa F. Albaqami, Haifa S. Almukadi, Ramu Elango, Noor Ahmad Shaik, Babajan Banaganapalli

**Affiliations:** ^1^ Department of Medical Laboratory Sciences, Faculty of Applied Medical Sciences, King Abdulaziz University, Jeddah, Saudi Arabia; ^2^ Princess Al-Jawhara Center of Excellence in Research of Hereditary Disorders, King Abdulaziz University, Jeddah, Saudi Arabia; ^3^ Centre of Artificial Intelligence for Precision Medicines, King Abdulaziz University, Jeddah, Saudi Arabia; ^4^ Department of Biological Sciences, Faculty of Science, King Abdulaziz University, Jeddah, Saudi Arabia; ^5^ Department of Laboratory Medicine, Faculty of Applied Medical Sciences, Umm Al-Qura University, Mecca, Saudi Arabia; ^6^ Department of Science, Prince Sultan Military College of Health Sciences, Dhahran, Saudi Arabia; ^7^ Department of Pharmacology and Toxicology, Faculty of Pharmacy, King Abdulaziz University, Jeddah, Saudi Arabia; ^8^ Department of Genetic Medicine, Faculty of Medicine, King Abdulaziz University, Jeddah, Saudi Arabia

**Keywords:** prostate cancer, microRNA, gene expression, PPI, cancer drug targets

## Abstract

**Background:** Prostate cancer (PC) is a fatally aggressive urogenital cancer killing millions of men, globally. Thus, this study aims to identify key miRNAs, target genes, and drug targets associated with prostate cancer metastasis.

**Methods:** The miRNA and mRNA expression datasets of 148 prostate tissue biopsies (39 tumours and 109 normal tissues), were analysed by differential gene expression analysis, protein interactome mapping, biological pathway analysis, miRNA-mRNA networking, drug target analysis, and survival curve analysis.

**Results:** The dysregulated expression of 53 miRNAs and their 250 target genes involved in Hedgehog, ErbB, and cAMP signalling pathways connected to cell growth, migration, and proliferation of prostate cancer cells was detected. The subsequent miRNA-mRNA network and expression status analysis have helped us in narrowing down their number to 3 hub miRNAs (hsa-miR-455-3p, hsa-miR-548c-3p, and hsa-miR-582-5p) and 9 hub genes (*NFIB, DICER1, GSK3B, DCAF7, FGFR1OP, ABHD2, NACC2, NR3C1*, *and FGF2*). Further investigations with different systems biology methods have prioritized *NR3C1, ABHD2,* and *GSK3B* as potential genes involved in prostate cancer metastasis owing to their high mutation load and expression status. Interestingly, down regulation of *NR3C1* seems to improve the prostate cancer patient survival rate beyond 150 months. The *NR3C1, ABHD2,* and *GSK3B* genes are predicted to be targeted by hsa-miR-582-5p, besides some antibodies, PROTACs and inhibitory molecules.

**Conclusion:** This study identified key miRNAs (miR-548c-3p and miR-582-5p) and target genes (*NR3C1, ABHD2,* and *GSK3B*) as potential biomarkers for metastatic prostate cancers from large-scale gene expression data using systems biology approaches.

## 1 Introduction

Prostate cancer is a urogenital cancer that accounts for 15% of all cancers in men globally (Group, UCSW, 2014; Van Gool and Pearson, 2014). PC is initially asymptomatic (Ratner, 2021), but painful urination, blood in the urine, or pelvic discomfort symptoms commonly arise as the disease progresses over the months and years (Screening and Board, 2002). The known risk factors for PC include increasing age, African-American ethnicity, fat rich diet, positive family history, and obesity (Bostwick et al., 2004). Early clinical testing and an accurate diagnosis of PC are critical in determining treatment options (Greenlee et al., 2001). The most common biomarker used to detect PC is prostate-specific antigen (PSA) test in the blood. However, it fails to distinguish indolent or aggressive cancer stages (Cancer, IAFRO, 2003). Besides this, increased age or an enlarged or inflamed prostate are the other factors that can also elevate serum PSA leading to misdiagnosis or unnecessary and costly treatment. This warrants the need to identify robust biomarkers that are not only specific and sensitive but also improve the overall accurate diagnosis and prognosis of prostate cancer.

Prostate cancer is estimated to have 58% heritability, highest among all major cancers (Lachance et al., 2018). Family-based linkage studies have identified a series of genes responsible for hereditary PC such as *HPC1* (Carter et al., 1992; Cooney et al., 1997; Berry et al., 2000a), *PCAP* (Neuhausen et al., 1999; Berry et al., 2000a; Xu et al., 2001), *HPCX* (Schleutker et al., 2000), *CAPB* (Berry et al., 2000a; Xu et al., 2001), *HPC20* (Berry et al., 2000b), *HOXB13* (Breyer et al., 2012; Xu et al., 2013) etc. Over the last decades, Genome wide associated studies (GWAS) have identified about 160 common risk loci in PC, suggesting a polygenic model of PC (Farashi et al., 2019). Most of these risk loci genes are involved in the cell cycle or DNA repair (*ATM, TERT, MYC,* and *MDM2*), inflammatory response (*IL8RB*), and metabolism (*JAZF1* and *HNFB*). However, under-representation of ethnic diversity necessitates us to look for universally applicable genetic susceptibility biomarkers for PC. Moreover, the molecular basis of PC could not be fully explained by candidate genetic variants alone, but through the global gene expression alterations. Some studies have confirmed the potential contribution of dysregulated gene expression changes in either blood (Wang et al., 1979; Papsidero et al., 1980; Kuriyama et al., 1981) or prostate biopsies (Ragde et al., 1988), and few were correlated with the survival rate of PC patients (Greenlee et al., 2001). These genes were mainly involved in diverse cellular functions like protein kinase binding, enzyme binding, cell activation and proliferation, *Wnt* regulation mechanisms, wound healing, apoptosis, etc. (Verras and Sun, 2006; Kypta and Waxman, 2012). However, the specific molecular regulators which control the gene expression changes have not been well characterized.

Gene expression is controlled at both post transcription and translation levels. MicroRNAs (miRNAs) are 18–22 bp long, and known to regulate more than 50% of protein-coding genes at post transcriptional level (Bartel, 2009). Few miRNAs have been identified as potential diagnostic biomarkers of tumour development and metastasis (Nikitina et al., 2012; Ilic et al., 2013). PC associated microRNAs in serum have the potential to successfully distinguish cancer and healthy controls. The aggressive PC is known to show over-expression of oncogenic miRNAs; miR-21, miR-125b, miR-221, and miR-222 (Sun et al., 2009). The miR-21 is over-expressed in PC and contribute to tumour growth (Nikitina et al., 2012) by knocking down *PTEN* and other tumour suppressor genes (Meng et al., 2007). The miR-200 family is regarded as key regulatory molecule in PC due to their down regulated expression tumor suppressive function and regulatory role in initiation and migration of prostate tumors (Feng et al., 2014). Furthermore, these 5 miRNAs when combined with routine PSA test were shown to improve the PC diagnosis (Siegel et al., 2020). However, influence of miRNAs on differentially expressed genes in prostate tissues is not explored in detail till now.

Owing to the sparse data, this study aimed to expand our current understanding of PC pathogenesis by exploring miRNA-mRNA interactions in prostate gland tissues. By involving a series of comprehensive bioinformatics approaches, this study has identified several gene-network clusters involved in cell communication, inflammation, proliferation, and differentiation processes which, are dysregulated in prostate cancers. Our findings provide a novel insight into understanding the mechanisms of PC, besides uncovering genetic markers with potential for disease diagnosis and therapeutic modulation.

## 2 Methodology

**FIGURE 1:**
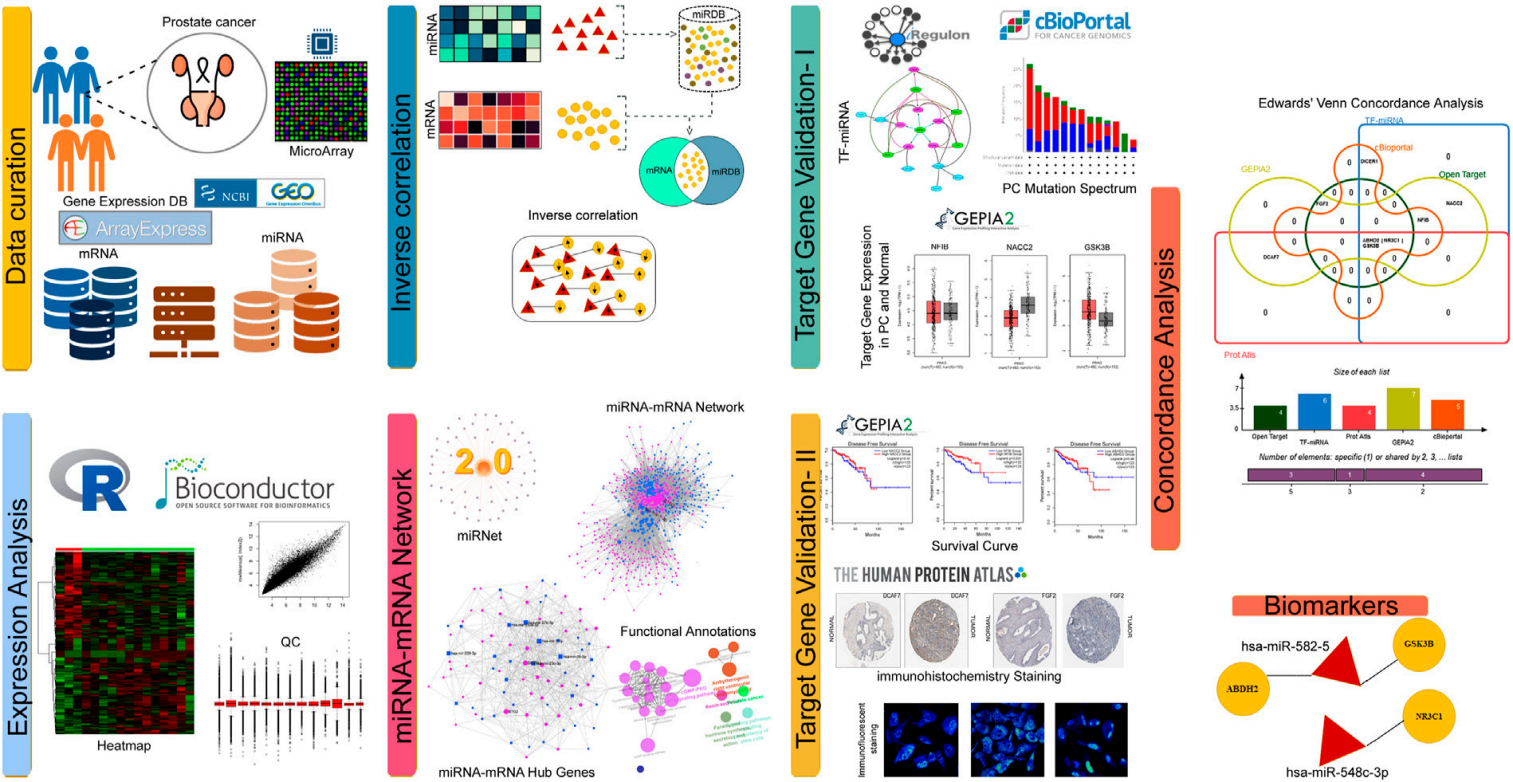
Infographics of PC mRNA-miRNA analysis.

The workflow of current work is presented in Figure 1.

### 2.1 Collection of prostate cancer mRNA and microRNA expression datasets

The NCBI-GEO and EMBL-EBI Array express databases were initially searched for PC gene expression datasets using keywords like “Prostate Cancer, mRNA, and miRNA.” From the output, one mRNA expression dataset, i.e., GSE6919, and one miRNA expression dataset, i.e., GSE21036, have been selected based on type and number of samples, data quality and expression array method used. The first dataset (GSE6919) consists of the gene expression data of 106 tissue samples (including 25 metastatic prostate tumours and 81 normal prostate tissues) generated on GPL8300 platform (Affymetrix human genome U95 version 2 array). The second dataset, GSE21036, consists of the expression data of miRNAs of 42 Samples (including 14 metastatic prostate tumours and 28 normal prostate tissues) generated on the GPL8227 platform (Agilent-019118 Human miRNA Microarray 2.0 G4470B (miRNA ID version).

### 2.2 Detection of differentially expressed genes and microRNAs in prostate cancer tissues

We performed differential expression analysis of miRNA and RNA profiles to find the prostate cancer specific dysregulations. We utilized GEO2R (https://www.ncbi.nlm.nih.gov/geo/geo2r/) webtool of NCBI to detect differentially expressed genes (DEGs) and differentially expressed miRNAs (DEMs) in the test datasets. The GEO2R webtool conducts comparative analysis on microarray expression data sets utilizing the GEOquery and limma R packages available in Bioconductor software. The genes or miRNAs showing 1.5-fold changes (FC) at adj. *p*-values of <0.01 were identified. The expression pattern of these miRNAs and mRNAs were graphically represented in the form of volcano and median mean difference plots.

### 2.3 Identification of potential target genes for DEMs

In the initial phase, miRNA IDs of the DEMs were used to search for their potential target genes in MiRDB webserver (http://mirdb.org/mirdb/mining.html). The target genes showing <70 prediction scores and miRNAs with >2000 predicted targets in the genome were excluded from the output data. Then, keeping the miRDB data as reference, the inverse correlation analysis between miRNA-mRNA expression statuses was performed to screen the miRNA-target gene pairs. The mathematical formula adopted for inverse-correlation of miRNA-mRNA levels is given below (Bima et al., 2022).

$$r=\frac{n\left(\sum xy\right)-\sum x\sum y}{\surd \left[n*\;\left(\sum x2\;–\;\left(\sum x\right)2\right)\right]\;*\;\left[n*\;\left(\sum y2\;–\;\left(\sum y\right)2\right)\right]}$$

In the formula, “n” represents the correlation coefficient, “X” represents DEGs and “Y” represents DEMs.

### 2.4 GO annotation of microRNA target genes

The miRNA target genes identified from the inverse correlation analysis were further explored with KEGG (Kyoto Encyclopaedia of Genes and Genomes, http://www.genome.jp/kegg/) pathway analysis using ClueGO v2.5., a cytoscape plug-in. KEGG links the genes to functional pathways, with a *p*-value of <0.05 as an enrichment cut-off measure (Kanehisa and Goto, 2000). ClueGO facilitates the visualization of functionally related genes as a clustered network and overview chart. The statistical test used for the enrichment was based on two-sided hypergeometric option with a Bonferroni step down *p*-value correction and functional leading group term based on their Kappa score (≥ 0.3).

### 2.5 Construction of microRNA-target gene transcriptome network

The prostate cancer genes and miRNA functional interactome network was constructed using miRNET webserver (http://www.mirnet.ca). The miRbase ID and Entrez/Ensembl gene ID were given as input options for miRNAs and DEGs, respectively. The output of this tool is a transcription network along with different network parameters like centrality, betweenness, shortest path etc. The constructed network was visualized through Cytoscape 3.7.1 software. From the network hubs - genes and miRNAs-were selected based on their highest centrality scores (Thul and Lindskog, 2018). Furthermore, the hypergeometric algorithm (option available in functional explorer plugin) was used to identify all KEGG pathways in which the hub genes were involved.

### 2.6 Functional assessment of microRNA target genes

In this step, the hub genes identified from miRNA-target gene pairs were further explored to identify their transcription factor (TF) motifs, and also to assess their drug tractability, expression status and mutational load.

#### 2.6.1 Construction of microRNA-Target gene-transcription factor network

The miRNA-target gene pairs from the network analysis were further explored in iRegulon plugin of Cytoscape to enrich their TF motifs (Janky et al., 2014). The motif prediction parameters were as follows, minimum orthologous gene identity of ≥0.05, the maximal false discovery rate (FDR) on motif similarity of ≤0.001, and normalized enrichment score (NES) of >3. Finally, the miRNA–mRNA-TF regulatory network was constructed utilizing Cytoscape 3.7.1.

#### 2.6.2 Therapeutic potential of hub genes

The hub genes from miRNA-target gene network were explored in open target platform (http://www.targetvalidation.org). The input option for this tool is query gene ID and the output includes phenotype association characteristics (*p* = <0.05), predicted tractability (small molecule inhibitors and antibodies) and known drug information (target diseases, mode of action, clinical trials etc.) about the query gene.

#### 2.6.3 Mutational load of hub genes in prostate cancers

All the query hub genes of miRNA-target network were explored in cBioPortal (http://www.cbioportal.org/) to assess their mutational load in prostate cancer tissues. Upon providing the gene ID and cancer type as an input data, oncoprint option available in this webserver visualises the genetic alterations of the queried genes in the form of heatmaps with z-score values. Additionally, the mutation pattern of hub gene pairs in prostate cancers is calculated with Log2 Odds Ratio (OR).

#### 2.6.4 Expression analysis of hub genes in prostate cancers

The expression status of query hub genes in normal and cancer tissues, and their disease-free survival curve correlation analysis was performed in GEPIA2 (http://gepia2.cancer-pku.cn/) webserver. The genes showing |log2 fold change| with ≥1 were considered as significant at the *p*-value of <0.01. Disease-free correlation of hub gene expression status between PC and normal tissues was determined using a log rank test with *p* < 0.05.

#### 2.6.5 Immunohistochemistry analysis

The expression status of hub genes across cell lines and tissues was estimated with Human protein atlas (https://www.proteinatlas.org/) database. This database intake the query gene or protein name and provides the immunocytochemistry/immunofluorescence (ICC-IF) information about the candidate protein’s subcellular location and expression (protein-transcripts per million, pTPM). pTPM, is a normalization method used for comparing the gene expression levels in the same tissue. We also analysed the immunohistochemistry data to enquire the tissue expression status of the query gene. Here the intensity of primary antibody staining between normal and tumour tissues was classified as negative, weak, moderate, or strong based on the detector grain settings used for image acquisition in combination with the visual appearance of the image.

## 3 Results

### 3.1 Identification of differentially expressed target genes and microRNAs

**FIGURE 2:**
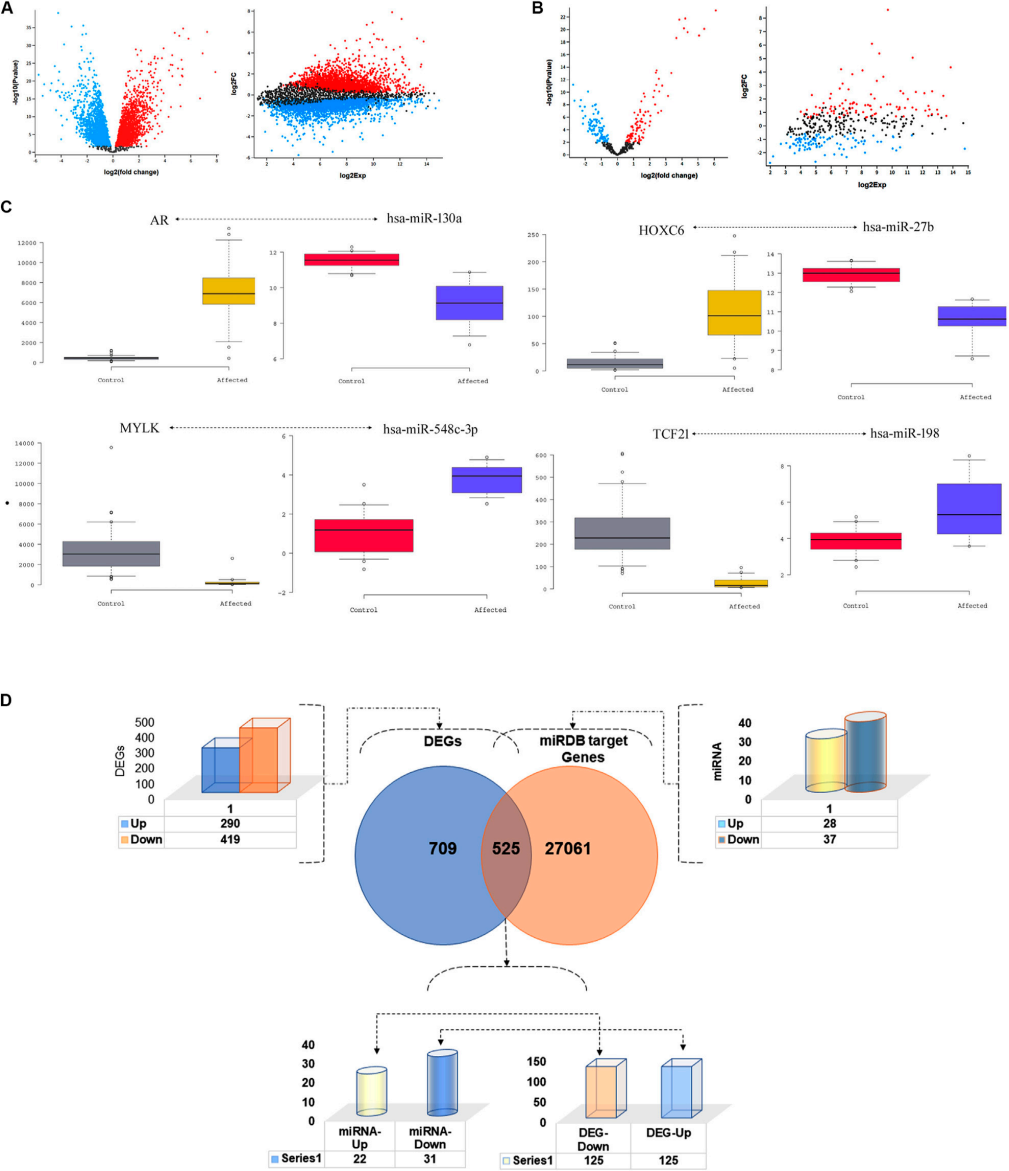
Distribution of differentially expressed genes and miRNAs. Volcano plots of **(A)** GSE6919 and **(B)** GSE21036 datasets showing the transcript expression profiles mRNA and miRNA respectively, of metastatic prostate cancer and normal prostate tissues. Red and blue dots correspond to the up and down regulated genes, respectively, showing more than 1.5-fold expression difference (*p* < 0.05). **(C)** Wesker Box plot of inverse co-related miRNA and their target DEGs **(D)**. DEGs and miRNA target gene filtration method.

The analysis of GSE6919 dataset has identified the expression of 9,153 genes corresponding to 12,558 probes. A total of 709 genes were found to be differentially expressed (log^2^ ≥ 1.5 folds), between metastatic prostate tissues compared to normal prostate tissues. Of these DEGs, 290 (40.90%) were upregulated and 419 (59.10%) were down regulated (*p* < 0.05). Simultaneously, a total of 373 miRNAs corresponding to 373 probes were detected from the analysis of GES21036 dataset. Metastatic prostate tumours have demonstrated the dysregulated expression (log^2^ ≥ 1.5 folds) of 65 miRNAs, including 28 upregulated and 37 downregulated when compared to normal prostate tissues (*p* < 0.05) (Figures 2A,B). The details of top 4 genes and miRNAs are listed in Table 1.

**TABLE 1:** The top four differentially expressed target genes and miRNAs.

Datasets	ID	Adj P.Val^1^	T^2^	B^3^	LogFC^4^	Gene symbol
GSE6919	34342_s_at	4.69E-18	11.231099	34.51877	4.8071724	*SPP1*
	1577_at	1.06E-40	23.506291	90.06834	3.8885371	*AR*
	32052_at	1.82E-23	13.81756	47.67757	3.6298491	*HBB*
	767_at	1.87E-47	−28.217604	106.65766	−6.2250421	*MYH11*
GSE21036	hsa-miR-548c-3p	1.67E-10	9.1171	16.9256	2.74741	hsa-miR-548c-3p
	hsa-miR-486-5p	3.50E-08	7.3722	11.0341	2.66815	hsa-miR-486-5p
	hsa-miR-133b	5.86E-19	−16.2568	37.2489	−5.38092	hsa-miR-133b
	hsa-miR-1	3.46E-21	−19.189	43.8432	−6.10077	hsa-miR-1

^1^Adjusted *p*-value; ^2^t-moderated t-statistic; ^3^B-statistic or log-odds that the gene is differentially expressed; ^4^Log2-fold change between two experimental conditions.

### 3.2 Mapping of microRNA target genes

The miRDB webserver predicted that, 53 miRNAs (of the 65 differentially expressed miRNAs) modulate the expression of 27,541 target genes at a cut off score of <70. Of these 27,541 target genes, 525 were overlapping with 709 DEGs detected in prostate metastatic tissues. The inverse correlation analysis of miRNA and DEG pair expression statuses have further narrowed down the total number of target genes to 250. Of this list, 125 downregulated genes were targeted by 22 upregulated miRNAs. The remaining 125 upregulated genes were modulated by 31 downregulated miRNAs. The top miRNA-target gene pairs identified in prostate cancer tissues is listed in Table 2; Figures 2C,D.

**TABLE 2:** The top miRNA-target gene pairs of prostate cancer tissues.

	#	miRNA	Target gene	Target score	Log Fc. miRNA^1^	Log Fc. DEGs
Up-Reg miRNA	1	hsa-miR-1225-5p	*ZNF516*	78	1.59574	−2.4506688
	2	hsa-miR-648	*MEIS2*	90	1.62921	−2.6731721
			*CXCL12*	80	1.62921	−1.5779949
	3	hsa-miR-602	*PFN1*	74	1.82483	−1.7949682
			*C16orf45*	76	1.82483	−1.660665
Down-Reg miRNA	1	hsa-miR-133b	*CAPN15*	96	−5.38092	1.5478958
	2	hsa-miR-99a	*GSK3B*	70	−2.5216	1.5973438
	3	hsa-miR-338-3p	*LDHA*	75	−1.61592	1.511398
			*CAMTA1*	80	−1.61592	1.7192758
			*UBFD1*	82	−1.61592	1.9780685

^1^Log Fc-log fold changes.

### 3.3 Pathway enrichment of microRNA target genes

The main KEGG pathways enriched for 250 targets genes (125 up and 125 downregulated genes, each) of 53 miRNAs at the *p*-value threshold of <0.05 are listed in Table 3. The downregulated genes were enriched in prostate cancer, cAMP signalling, signalling pathways regulating pluripotency of stem cells, Th1 and Th2 cell differentiation, and cGMP-PKG signalling pathways. The upregulated genes were enriched in Hedgehog signalling, ErbB signalling, and T-cell receptor signalling pathways.

**TABLE 3:** KEGG pathways enriched for miRNA target genes of prostate cancer tissues.

DEG dysregulation	KEGG ID	Term\Pathway	Bonferroni *p*-value	% Associated Genes	Target genes of miRNAs
Upregulated DEGs	KEGG:04,340	Hedgehog signalling pathway	0.03	5.36	*CSNK1G2, GRK3, GSK3B*
	KEGG:04,012	ErbB signalling pathway	0.03	4.71	*CBLB, CRKL, GSK3B, PAK2*
	KEGG:04,660	T-cell receptor signalling pathway	0.01	4.81	*CBLB, GSK3B, NFATC1, PAK2, PPP3CA*
Downregulated DEGs	KEGG:05,215	Prostate cancer	0.04	4.12	*FGFR2, IGF1, KLK3, ZEB1*
	KEGG:04,550	Signalling pathways regulating pluripotency of stem cells	0.02	4.20	*BMPR1A, FGF2, FGFR2, FZD7, ID4, IGF1*
	KEGG:04,658	Th1 and Th2 cell differentiation	0.05	4.35	*FOS, GATA3, PPP3CB, STAT6*
	KEGG:04,022	cGMP-PKG signalling pathway	0.00	5.99	*ATP1A2, ATP1B1, ATP2A2, ATP2B4, EDNRA, KCNMB1, MYLK, PDE5A, PLCB1, PPP3CB*
	KEGG:04,024	cAMP signalling pathway	0.00	4.17	*ATP1A2, ATP1B1, ATP2A2, ATP2B4, BDNF, EDNRA, FOS, PDE4B, PDE4D*

### 3.4 MicroRNA-target gene interaction network analysis

**FIGURE 3:**
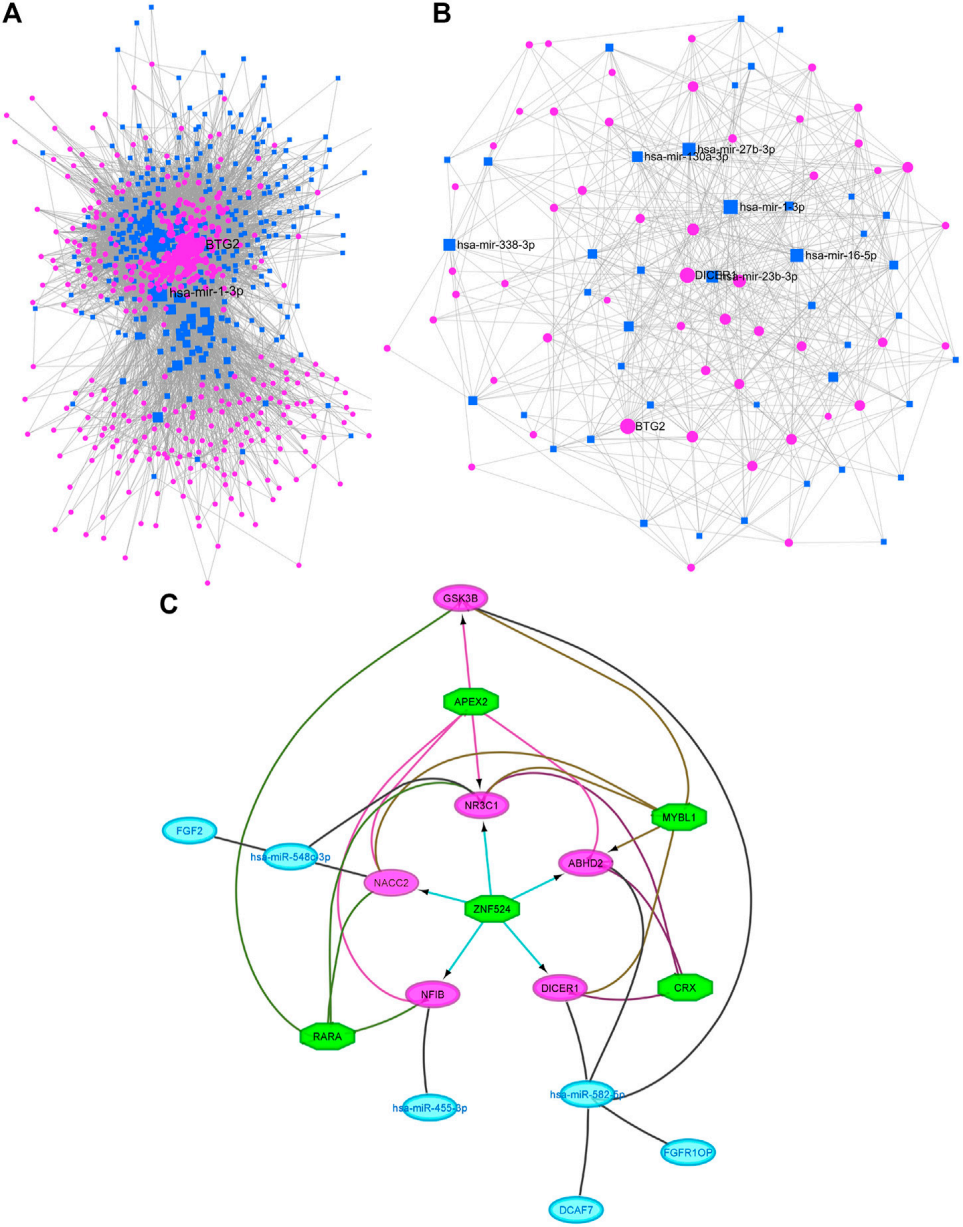
The miRNA-Protein interaction network of prostate cancer tissues. **(A)** The overall network of miRNAs and genes. **(B)** miRNA (Square)and their target hub genes (circle)with degree of centrality (>150). **(C)** Regulatory networks of the miRNAs, target genes and transcription factors. Green octagons represent TFs. Purple circles represent target genes regulated by transcription factors and key miRNAs (blue circles).

Molecular networks highlight the physical contacts among protein partners. They are critical in most basic molecular mechanisms involved in cellular function but are often perturbed in disease states. The miRNA-mRNA network of 250 target genes and 53 miRNAs consisted of 9,931 nodes and 34,474 edges (Figure 3A). Considering the network parameters at >150 centrality and betweenness of >254320.9 as filtration criteria, we identified hub genes, of which 32 are target genes and 6 miRNAs (hsa-miR-455-3p, hsa-miR-671-5p, hsa-miR-548c-3p, hsa-miR-125a-3p, hsa-miR-338-3p, and hsa-miR-582-5p) (Figure 3B).

The functional enrichment of these hub miRNA-target gene pairs has confirmed the strong interaction existing between them. Of the 32 target hub genes, 4 were mapped to cell signalling pathways including *ACVR1* and *ACVR2B* in TGF-beta signalling, *CDKN1A* and *GSK3B* in ErbB signalling (adj.P val = 0.016) and *CDKN1A* and *GSK3B* genes in Prostate cancer pathways (adj. P val = 0.076). *CDKN1A* and *GSK3B* were mapped to Cell cycle pathways as well (adj. P val = 0.02). *ACVR1* and *ACVR2B* are also playing a role in Cytokine-cytokine receptor interaction pathway (adj.P val = 0.062) (Supplementary Table S1). Out of the 6 miRNAs, only 3 (hsa-miR-455-3p, hsa-miR-548c-3p, and hsa-miR-582-5p) were found to target 9/32 (28.1%) genes, while the remaining 3 (hsa-miR-671-5p, hsa-miR-125a-3p, and hsa-miR-338-3p) do not have any targets in the hub genes. So, those three were eliminated from further analysis. The down-regulated hsa-miR-455-3p modulates *NFIB* gene (upregulated), and hsa-miR-582-5p targets *DICER1, GSK3B, DCAF7, FGFR1OP,* and *ABHD2* genes (upregulated), while upregulated hsa-miR-548c-3p targets *NACC2, NR3C1 and FGF2* genes (downregulated).

### 3.5 Systems biology validation of prostate cancer hub genes

We performed the functional validation of the 9 hub genes (with inverse regulated miRNAs mentioned above) in the prostate cancers, using different systems biology approaches.

#### 3.5.1 Identification of TF and microRNA target gene network regulators

The enrichment findings of TF motifs for nine hub target genes and six miRNAs have predicted that the transcription factors like *RARA, CRX, ZNF524, APEX2, and MYBL1* interacts with 6 hub genes, like *ABHD2, DICER1, GSK3B, NACC2, NFIB,* and *NR3C1* (with network enrichment score of >3.5) (Table 4). Although, the miRNAs were not directly connected to TF motifs, but three miRNAs were predicted to regulate the network of TF-target gene network. For example, hsa-mir-548c-3p interacts with *NACC2* and *NR3C1*, hsa-mir-455-3p interacts with *NFIB,* and hsa-mir-582-5p interacts with *ABHD2* and *DICER1* (Figure 3C).

**TABLE 4:** The network analysis findings of transcriptional factors, and their motifs with miRNA-target genes.

miRNA	Motif ID	AUS^1^	NES^2^	Transcription factor	Target genes
hsa-miR-455-3p	taipale-ACCCTTGAACCC-ZNF524-full	0.434978	5.45039	*ZNF524*	*ABHD2, NACC2, NFIB, DICER1, NR3C1*
	hdpi-RARA	0.379007	4.61005	*RARA*	*GSK3B, NR3C1, NACC2, NFIB*
	hdpi-APEX2	0.341139	4.04151	*APEX2*	*GSK3B, NFIB, ABHD2, NACC2, NR3C1*
hsa-miR-548c-3p	yetfasco-1433.1	0.361236	4.34324	*MYBL1*	*DICER1, ABHD2, GSK3B, NR3C1, NACC2*
	transfac_public-M00004	0.307922	3.54279	*MYBL1*	*NACC2, NR3C1, DCAF7*
has-miR-582-5p	tfdimers-MD00087	0.337652	3.98915	*CRX*	*NR3C1, ABHD2, DICER1*
	transfac_public-M00004	0.307922	3.54279	*MYBL1*	*NACC2, NR3C1, DCAF7*

^1^Area Under the cumulative recovery curve, ^2^Normalized Enrichment Score.

#### 3.5.2 Molecular tractability potential of hub genes

The nine hub genes (9/32; 28%) selected from the topological analysis demonstrated the genotype-phenotype association score of >0.002 in the Open Target Validation Platform analysis. Tractability information for all 9 queried genes was available, with *FGF2*, *GSK3B,* and *NR3C1* genes were tractable by small molecules, *ABHD2*, *FGF2,* and *GSK3B* genes were tractable by antibody molecules, *ABHD2, FGF2, NR3C1,* and *GSK3B* genes were targeted by Proteolysis Targeting Chimeras (PROTACs), while only *FGF2* gene was tractable also by enzyme molecules (Table 5). Of note, the *FGF2* acts as a molecular target for Muprafostat oligosaccharide agent, which is currently in phase 2 trial in prostate cancer. The *GSK3B* is targeted by lithium carbonate inhibitor which currently completed phase 1 trial in prostate cancer. The *NR3C1* is targeted by several small molecule agonists like Dexamethasone, Prednisolone, and Prednisone which have currently completed phase 4 clinical trials (Supplementary Table S2).

**TABLE 5:** Open Target Platform output for disease association, known drugs, action and clinical trial, tractability of 9 hub genes.

Gene	Geno-pheno association	Known drugs	Action	Clinical trial phase	Tractability predictions			
					Small molecule clinical precedence	Antibody clinical precedence	Protac	Other modalities (enzyme, peptide, oligonucleotide etc.)
*ABHD2*	15	—	—	—	—	+ +	+	—
*FGF2*	296	Muparfostat	Inhibitor	>1	+3	+ +	+3	+
*DCAF7*	16	—	—	—	—	—	+ +	—
*GSK3B*	199	Lithium Carbonate	Inhibitor	>1	+3	+ +	+3	—
		Ly-2090314	Inhibitor	>1				
		9-Ing-41	Inhibitor	1				
*NACC2*	13	—	—	—	—	—	+ +	—
*NFIB*	148	—	—	—	—	—	+ +	—
*DICER1*	306	—	—	—	—	—	+ +	—
*NR3C1*	265	Dexamethasone	Agonist	>1	+3	—	+3	—
		Prednisolone	Agonist	>1				
		Prednisone	Agonist	>0				
		Hydrocortisone	Agonist	>1				
		Cyproterone acetate	Antagonist	>2				
		Methylprednisolone	Agonist	>2				
		Mifepristone	Antagonist	>1				
		Relacorilant	Antagonist	1				
*FGFR1OP (CEP43)*	75	—		—	—	—	+	—

#### 3.5.3 Determination of mutation load of hub genes

**FIGURE 4:**
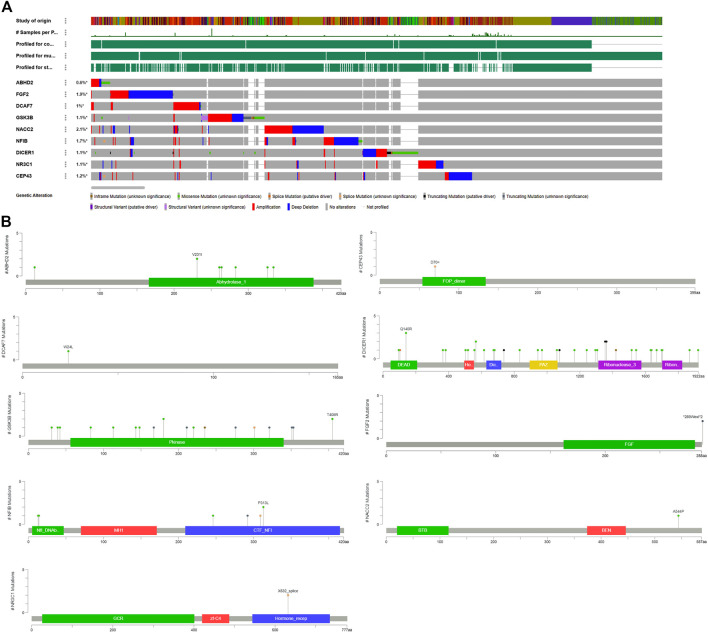
Mutation load of hub genes. **(A)** Distribution and frequency of genetic alternations in PC hub genes. **(B)** The “lollipop” plot generated by the Mutation Mapper tool of cBioPortal shows the open box of the nine hub genes, as well as the frequency, position, and the domain of mutations in the chromosome.

The cBioPortal for Cancer Genomics analysis has confirmed that all the hub genes carry different types of alterations, including in frameshift, missense, splice, and truncating mutations, structural variants, amplifications, and deep deletions (Figure 4A). Of all the genes, *NACC2* gene alterations were reported in 105/4990 (2.1%) prostate cancer samples. The other genes showing molecular alterations in prostate cancer samples from highest to lowest are as follows; *FGF2* (94/4990; 1.9%), *NFIB* (86/4990; 1.7%), *CEP43* (62/4990; 1.2%), *GSK3B* (99/8961; 1.1%), *DICER1* (101/8961; 1.1%), *NR3C1* (53/4990; 1.1%) and *ABHD2* (28/4,990; 0.6%) (Supplementary Table S3). Interestingly, in 5/9 genes (55.5%), i.e., *ABHD2, DICER1, GSK3B, NR3C1,* and *NFIB* mutations were localized to functional domains of their corresponding proteins, which suggest them to be highly critical genes for prostate cancers (Figure 4B). The mutations in NFIB gene are also co-occur with *NACC2, DCAF7* and *FGF2* genes (OR is three; *p* < 0.001) (Table 6).

**TABLE 6:** Co-occurrence analysis of hub genes of prostate cancers*.

A	B	Neither	A not B	B not A	Both	Log2 odds ratio	*p*-value	q-Value	Tendency
NACC2	NFIB	4,303	92	71	15	>3	<0.001	<0.001	Co-occurrence
DCAF7	NACC2	4,335	39	97	10	>3	<0.001	<0.001	Co-occurrence
NFIB	DICER1	4,342	76	53	10	>3	<0.001	<0.001	Co-occurrence
ABHD2	CEP43	4,395	22	58	6	>3	<0.001	<0.001	Co-occurrence
DCAF7	CEP43	4,375	42	57	7	>3	<0.001	<0.001	Co-occurrence
NFIB	CEP43	4,339	78	56	8	2.99	<0.001	<0.001	Co-occurrence
ABHD2	DCAF7	4,408	24	45	4	>3	<0.001	0.001	Co-occurrence
FGF2	DICER1	4,331	87	56	7	2.638	<0.001	0.001	Co-occurrence
FGF2	NFIB	4,309	86	78	8	2.361	<0.001	0.002	Co-occurrence
DICER1	NR3C1	4,372	58	46	5	>3	<0.001	0.002	Co-occurrence
NACC2	CEP43	4,317	100	57	7	2.406	<0.001	0.002	Co-occurrence
NACC2	NR3C1	4,329	101	45	6	2.515	0.001	0.004	Co-occurrence
GSK3B	DICER1	8,756	101	95	6	2.453	0.001	0.004	Co-occurrence
ABHD2	NFIB	4,371	24	82	4	>3	0.002	0.005	Co-occurrence
DICER1	CEP43	4,358	59	60	4	2.3	0.012	0.029	Co-occurrence
DCAF7	NFIB	4,350	45	82	4	2.237	0.014	0.029	Co-occurrence
ABHD2	GSK3B	4,374	25	79	3	2.732	0.014	0.029	Co-occurrence
NFIB	NR3C1	4,348	82	47	4	2.174	0.016	0.032	Co-occurrence
DCAF7	NR3C1	4,384	46	48	3	2.574	0.018	0.034	Co-occurrence

*Generated in cBioportal.

#### 3.5.4 Confirmation of expression status of hub genes in prostate cancer tissues

**FIGURE 5:**
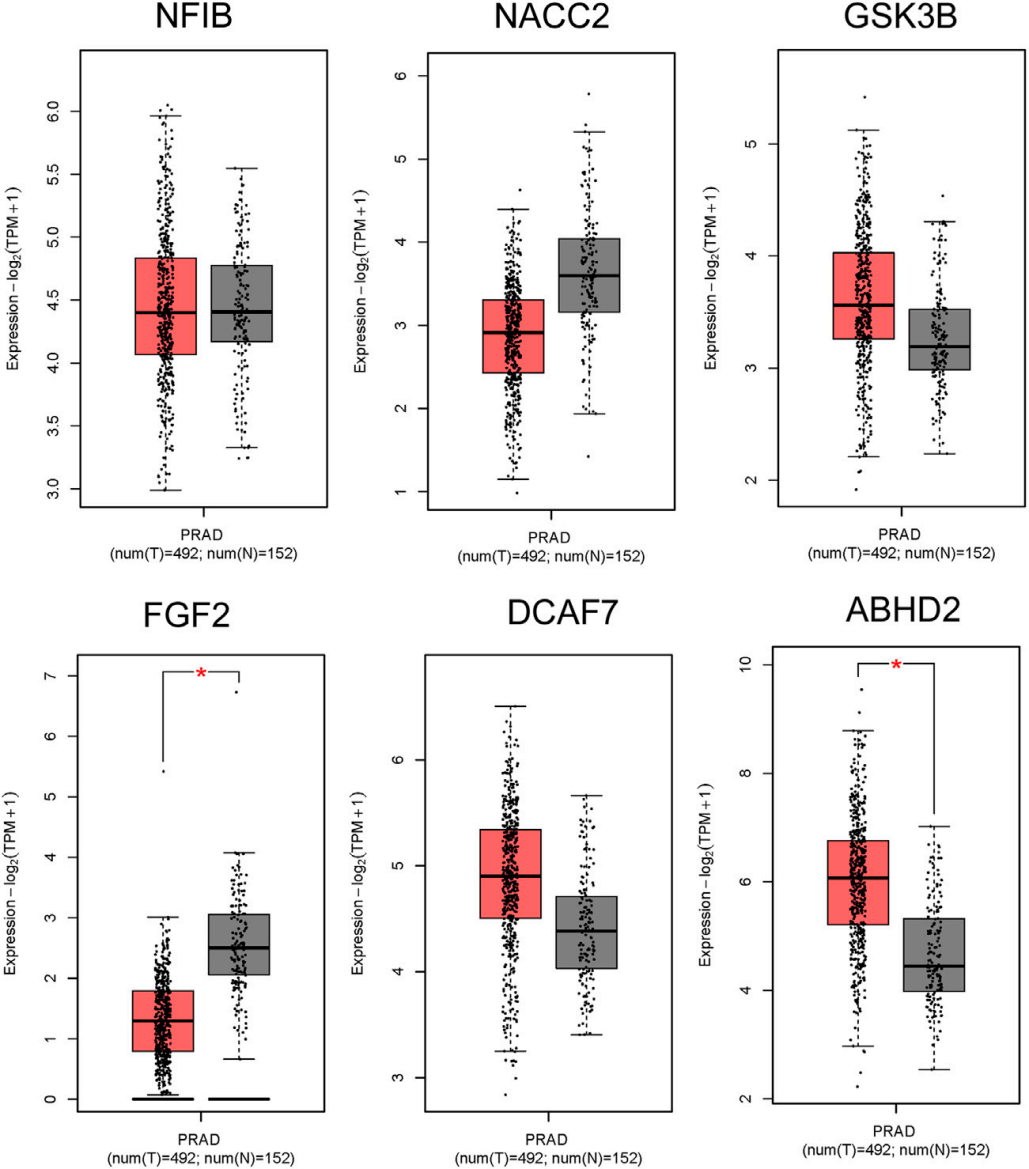
RNA Expression levels in PC in comparison to normal tissues from GEPIA2. The box plot representation for *ABHD2, FGF2, DCAF7, GSK3B, NACC2, and NFIB*. The signature score is calculated by mean value of log2 (TPM +1). The |Log2FC| cut-off of the expression of proposed biomarker was 1. The significant cut-off *p*-value of the expression of proposed biomarker was 0.01. The red box indicates the tumor samples while grey colour indicates the normal tissues. Each dot represents one sample data in the category.

The GEPIA results of all the 9 hub genes, have showed that expression levels of *ABHD2, DCAF7,* and *GSK3B* were upregulated, *FGF2, NACC2,* and *NR3C1* were downregulated in prostate adenocarcinomas (PRAD) than the normal prostate tissues by more than 1.5 folds (*p* < 0.01), reinforcing our understanding that these genes have important role in carcinogenesis (Figure 5). On the other hand, *DICER1* and *FGFR1OP* showed contradictory results to GEPIA analysis and the *NFIB* was non-significant.

#### 3.5.5 The prognostic value of hub genes in prostate cancer patients

**FIGURE 6:**
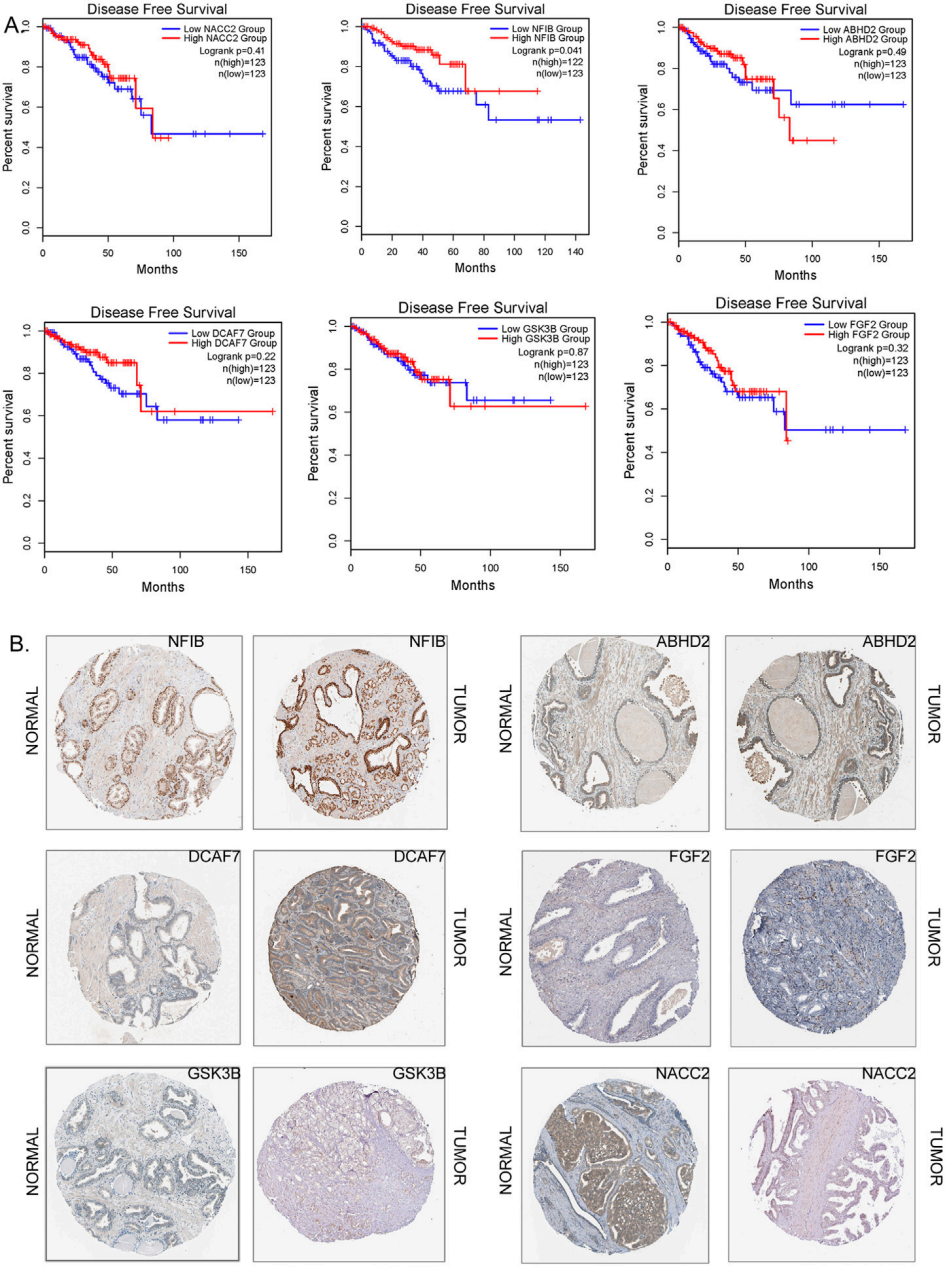
**(A)** The Kaplan-Meier survival curves as prognostic values (disease survival in days) of the hub genes (NACC2 Logrank *p* = 0.41; NFIB Logrank *p* = 0.041;ABDH2 Logrank *p* = 0.49; DCAF7 Logrank *p* = 0.22; GSK3B Logrank *p* = 0.87; FGF2 Logrank *p* = 0.32) of prostate cancer. The correlation of survival status of patients with different prostate cancer subtypes. **(B)** Immunohistochemistry of hub genes in prostate cancers and in normal prostate tissues (image extracted from https://www.proteinatlas.org/).

Kaplan-Meier survival curves showed that none of the 9 hub genes had any positive impact on improving overall survival of prostate cancer patients. However, the expression levels *NFIB* and *NR3C1* were indicated to be of predictive for disease-free survival (Figure 6A). The high expression level of *NFIB* could indicate poor survival rate in more than 50% of prostate cancer patients (*p* < 0.05). On the other hand, low expression level of *NR3C1* could extend the disease-free survival rate in 45% of prostate cancer patients beyond 150 months (*p* < 0.05). In comparison, there were no obvious associations between the expression levels of *ABHD2, FGF2, DCAF7, GSK3B, NACC2, DICER1,* and *FGFR1OP* genes and survival rate in prostate cancer patients.

#### 3.5.6 Immunocytochemistry/immunofluorescence analysis

**FIGURE 7:**
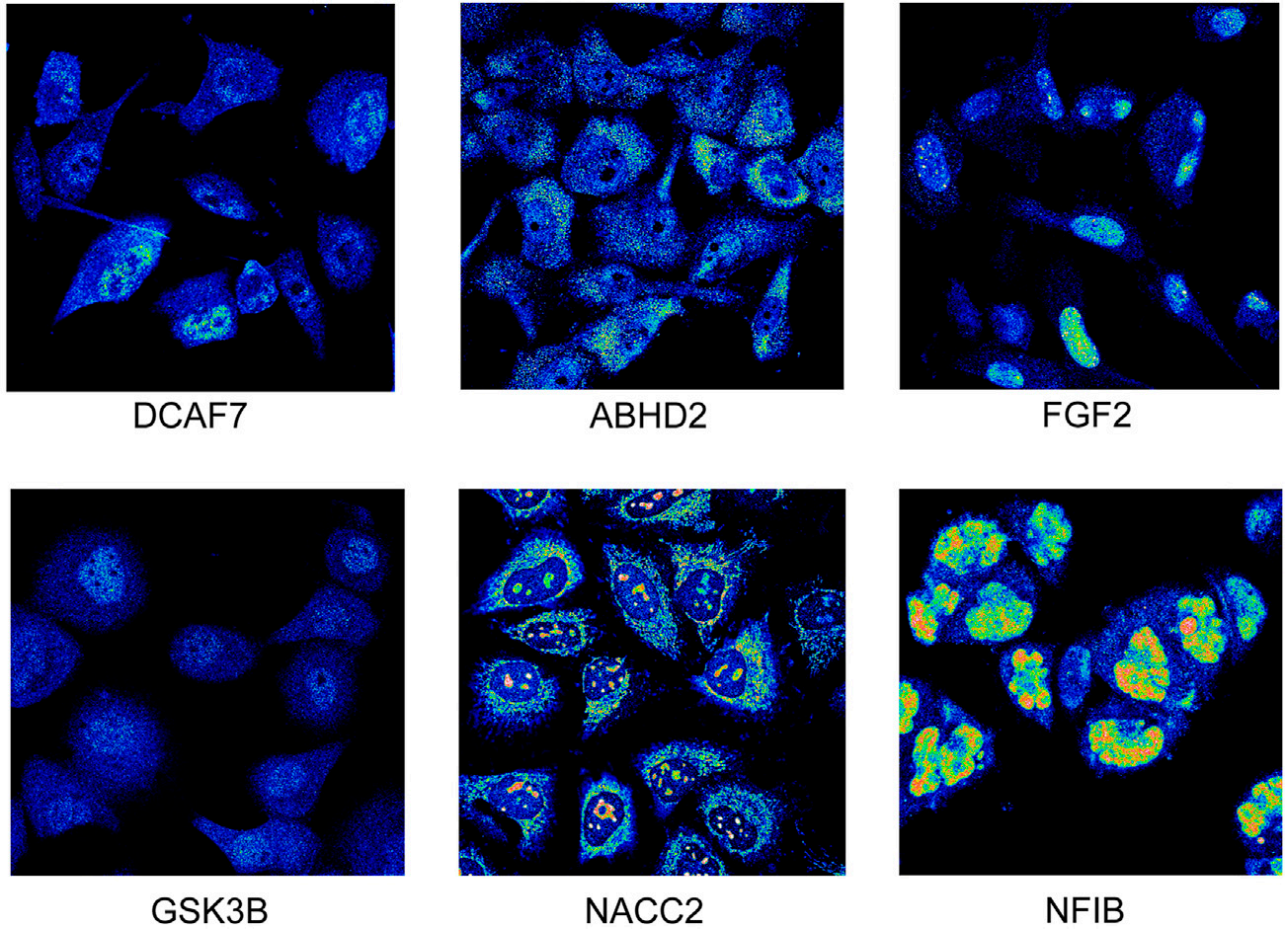
Immunofluorescence staining. The expression of prostate cancer hub genes in different cellular locations. (image extracted from https://www.proteinatlas.org/).

The protein expression of 6 hub genes in prostate cancer tissues are presented in Figure 6B. The results showed that *ABHD2, DCAF7,* and *GSK3B* were upregulated in cancer tissues compared with normal prostate tissues with high or medium intensity. However, the expression of *DICER1, FGF2, NACC2, NFIB, FGFR1OP,* and *NR3C1* in PC tissues shown low intensity. The HPA indirect immunofluorescence analysis provides the subcellular localization of hub genes in different cell compartments in different human cancer cell lines. Our results showed that majority hub genes *DCAF7, FGF2, GSK3B, NACC2,* and *NFIB* were highly expressed and (nTPM >19) localised mainly in the nuclei of the cancer cells. While *ABHD2,* and *DICER1* genes are mainly expressed (>18 nTPM) in the cytosol. Interestingly, the expression of *NR3C1* is seen in both nuclear and cytosolic compartments (Supplementary Table S4; Figure 7). Our findings provide spatial information on protein expression patterns of hub genes and define the subcellular localization to cellular organelles and structures at a single cell level. In summary, our results indicated that protein expression levels of 9 hub genes are dysregulated in prostate cancers compared to normal prostate tissues.

### 3.6 Concordance analysis

**FIGURE 8:**
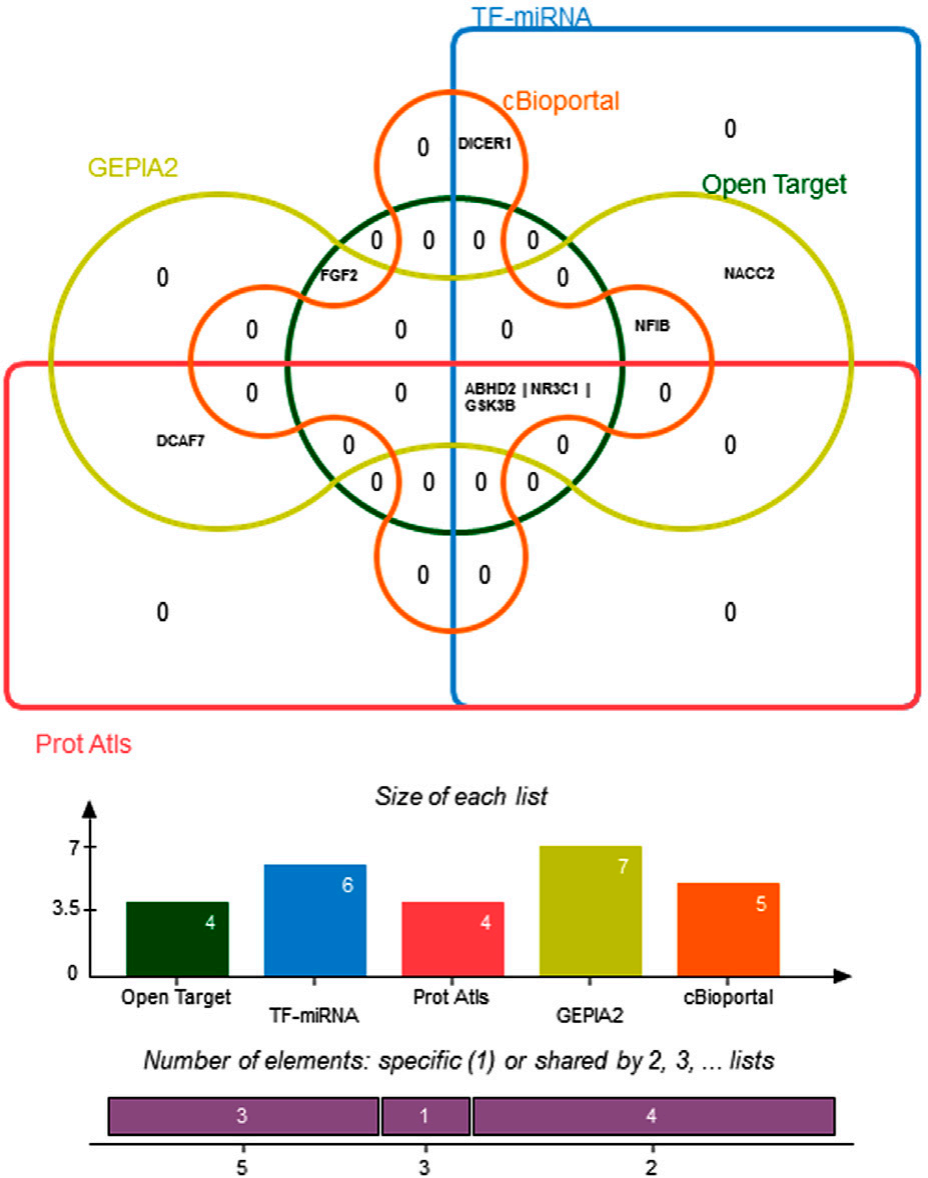
Edwards’ Venn diagram demonstrating overlapping of hub genes across six system biology analysis methods. The bar graph represents the number of genes in each prediction tool.

Edward Venn diagram in Figure 8 represent a concordance analysis of nine hub genes and five miRNA using different paradigm system biology methods. In the six functional prediction tools, the genes *ABDH2*, *NR3C1*, and *GSK3B* are significantly enriched. Interestingly, TF-miRNA analysis revealed an indirect connection between hsa-miR-582-5 and hsa-miR-548c-3p and TF. These miRNAs have the potential to regulate *ABDH2*, *NR3C1*, and *GSK3B*.

## 4 Discussion

Prostate cancer has a high clinical heterogeneity, ranging from fairly indolent to a fatally aggressive form (Litwin and Tan, 2017). This necessitates the need to search for novel prostate tumour biomarkers that can improve the molecular diagnosis and clinical outcomes. Several investigations have identified unique molecular subtypes of primary prostate tumors as well as important genetic changes that contribute to metastasis (Chen et al., 2021) (Zhao et al., 2021) (De Schaetzen Van Brienen et al., 2021). But these studies differ from each other in sample type, study design, statistical measures, and systems biology methods used. Moreover, the data on the role of miRNAs in regulating gene expression changes in prostate cancer tissues is sparce (Doghish et al., 2021) (Gordanpour et al., 2012) (Watahiki et al., 2011). Therefore, this new study design combined the publicly available expression datasets from different prostate tissues and across different microarray platforms to precisely map the interactions of key genes and miRNA involved in prostate tumorigenesis.

By normalization of expression data and comparison to normal prostate tissues, we detected 250 differentially expressed genes (125 up- and 125 downregulated genes) in the prostate cancer tissues. Pathway enrichment of these key upregulated genes highlight their involvement in Hedgehog signalling and ErbB signalling pathways. The Hedgehog (Hh) signalling network plays an important role in metastatic human prostate tumours that expressed Hh ligands and Hh-target genes at higher levels (Karhadkar et al., 2004). Moreover, chemical inhibitors of Hh signalling have been demonstrated to suppress the growth of PC cell lines (Sanchez et al., 2005). ErbB-2 oncogene contributes to PC progression by regulating numerous signalling pathways, including AKT, ERK1/2 and STATs, by positively influencing cell survival, migration, and proliferation of cancer cells (Miller et al., 2019).

The downregulated genes were enriched in key prostate cancer pathway (KEGG:05,215), which regulates growth factor receptor mediated cytokine-cytokine interactions (Lee and Rhee, 2017). These growth factor receptors indirectly activate the RAS-MAPK signalling that regulates gene expression, cellular growth, and survival of prostate cancer cells (Rodríguez-Berriguete et al., 2012). Increased cell proliferation and resistance to apoptosis may be caused by abnormal MAPK signaling pathway (Dasgupta et al., 2020). cAMP serves as a intermediary messenger molecule in several signaling pathways such as cell growth and differentiation (Kim et al., 2005). In the context of PC, cAMP levels modulate the activity of androgen receptor (AR) signalling, which could negatively impact normal prostate development and function (Yan et al., 2016).

We also identified miRNA alterations targeting genes which are regulating the transition of normal prostate to aggressive cancerous cells. The hub gene is a gene with the highest degree of connectivity (at top 10%) in the key module and identifying the hub genes is shown to improve the molecular dissection of human diseases like obesity (Sabir et al., 2019), autoimmune diseases (Banaganapalli et al., 2020), myocardial infarctions (Mujalli et al., 2020), and cancers (Sahly et al., 2021). So, by constructing the miRNA-mRNA functional network of 250 target genes and 53 miRNAs, we mapped 32 hub target genes and six hub miRNAs showing high degree of network centrality parameter (>150).

The inverse correlation analysis of expression statuses of these target gene-miRNA pairs has narrowed down the total hub genes to 9 (*NFIB, DICER1, GSK3B, DCAF7, FGFR1OP, ABHD2, NACC2, NR3C1, and FGF2*) and hub miRNAs to 6 (hsa-miR-455-3p, hsa-miR-548c-3p, hsa-miR-582-5p, hsa-miR-671-5p, hsa-miR-125a-3p and hsa-miR-338-3p). Among the hub miRNAs exclusively deregulated in PC, only 3 miRNAs (hsa-miR-455-3p, hsa-miR-548c-3p and hsa-miR-582-5p) were found to target 9 hub genes. The pathway analysis of these hub genes and miRNAs showed their involvement in different pathways connected to cell division or differentiation in response to extracellular signals, and a variety of cellular processes including gene expression, regulation of growth proteins, cell proliferation, oncogenic transformation, cell migration, and membrane trafficking in cancers (Kim et al., 2005). Thus, our findings broadly agree with previous studies, which highlighted differential regulation of key genes and miRNAs involved in the transformation of normal prostate tissue to prostate cancers (Gordanpour et al., 2012) (Chen et al., 2021) (Zhao et al., 2021) (De Schaetzen Van Brienen et al., 2021).

Both miRNAs and TFs regulate the gene expression at post-transcriptional and post-translation levels, respectively. Interestingly, miRNAs and TFs can regulate each other and also co-regulate a common target gene by forming a feed-forward loop (FFL) unit, which further forms gene regulatory networks (Qin et al., 2020). In this study, three miRNAs were predicted to co-regulate the network of TF-target gene network. For example, hsa-mir-548c-3p is shown to coregulate the interaction of *NACC2* and *NR3C1* with TF-ZNF524; hsa-mir-455-3p co-regulates the interaction of *NFIB* with TF-ZNF524, and hsa-mir-582-5p coregulate the interaction of *ABHD2* and *DICER1* with *TF-ZNF524* and *TF-CRX*.

The comprehensive systems biology validation of 9 hub genes has revealed that downregulated miRNA like hsa-miR-582-5p is correlated with the elevated the expression of *ABHD2* and *GSK3B* genes in prostate cancer tissues, and upregulated miRNA like hsa-miR-548c-3p correlates with the lower expression of *NR3C1* (glucocorticoid receptor) gene. The hsa-miR-582-5p inhibits metastasis of prostate cancer by repressing multiple components of TGF-β signalling, resulting in the inactivation of TGF-β signalling (Gordanpour et al., 2012) (Huang et al., 2019). Of note, miR-548c-3p acts as regulator in many cancers by significantly affecting both ErbB and Hippo signalling pathways (Feng et al., 2019). Hence, both miRNAs can be potential biomarkers for metastatic prostate cancers. Open target analysis findings have predicted that, Proteolysis Targeting Chimeras (PROTACs) can target all *ABHD2, GSK3B,* and *NR3C1* genes. Additionally, *ABHD2* is targeted by antibody molecules, and *GSK3B* is targeted by lithium carbonate inhibitor which has currently completed phase 1 trial in prostate cancer. *NR3C1* agonists like prednisone and dexamethasone are already under usage in lymphoid cancers and can be explored as potential repurposed drug for prostate cancers.

The *GSK3B*, a central component of PI3K/Akt survival pathway, determines the cancer cell survival through its effects on apoptosis. Expression status of *GSK3B* is positively correlated with tumor progression in multiple human malignancies (Li et al., 2015). Immunohistochemistry analysis of 499 PC surgical specimens showed higher levels of cytoplasmic (not nuclear) of GSK3B protein, in addition to its correlation with aggressive clinicopathological parameters such as late clinical stage, lymph node metastasis, extracapsular extension, and a high Gleason score, as well as a 2-fold reduction in recurrence-free survival with up to 12-years follow-up (Li et al., 2009). Since it is an androgen-regulated gene that is associated with the development of prostate cancer and its ability to resist chemotherapy, *ABHD2* is a promising new target for prostate cancer screening and treatment. Gleason score, pathological node stage, low cancer-specific survival rates, and resistance to docetaxel-based chemotherapy have been linked to *ABHD2* levels in prostate cancer specimens by immunohistochemical analysis. (Obinata et al., 2016). The *NR3C1*encodes a nuclear hormone receptor, acting as a transcription factor and modulate the gene expression across different tissues (Gu et al., 2017). Lower *NR3C1* expression levels were found in cancer cells compared to normal tissues of breast (Mamoor, 2021). For lymphoid cancers, acute lymphoblastic leukemia, chronic lymphocytic leukemia and multiple myeloma, *NR3C1* activation has been proven to be an effective treatment strategy. There are, however, several lines of evidence suggesting that GR activation has a strong effect on cancer cell behaviours such as invasion, apoptosis resistance and growth (Prekovic et al., 2021). Thus, it is imperative to use therapeutic interventions, whether agonists or antagonists to control the prostate carcinogenesis by regulating the expression of concerned candidate genes.

This study would like to sincerely clarify some limitations. First, we studied the role of miRNA and target expressions in metastatic prostate cancers. Thus, we do not generalise our findings neither blood samples nor primary tumors of prostate cancer patients. Second, we took microarray data of prostate tissue biopsies. Thus, mapping the key miRNAs or genes in body fluids like blood, urine and prostatic fluid is important to develop non-invasive biomarkers for prostate carcinogenesis. Third, the key hub genes and hub miRNAs identified in this study, need to be further validated by targeted approaches like real-time PCR, functional biology assays and on larger number of patient samples.

In conclusion, this study identified the inversely correlated dysregulation of 9 target genes (*NFIB, DICER1, GSK3B, DCAF7, FGFR1OP, ABHD2, NACC2, NR3C1,* and *FGF2*) and three key miRNAs (hsa-miR-455-3p, hsa-miR-548c-3p and hsa-miR-582-5p). This study supports the robustness of computational concepts, like protein interactome mapping, functional enrichment of biological pathways and construction of miRNA-mRNA and transcription factor gene networks in identifying prostate cancer biomarkers from large-scale gene expression data. Tissue based prostate cancer markers can be correlated with cancer development, which might offer early clinical interventions and therapy. This study also provides the initial evidence for the future knowledge driven functional studies of these miRNAs and their gene targets in prostate cancer.

## Data Availability

The datasets presented in this study can be found in online repositories. The names of the repository/repositories and accession number(s) can be found in the article/Supplementary Material.
