# Supplementary figures and images for: A comparative mRNA- and miRNA transcriptomics reveals novel molecular signatures associated with metastatic prostate cancers
Source: Front Genet. 2022 Nov 16;13:1066118. doi: 10.3389/fgene.2022.1066118 (PMC9708707; doi:10.3389/fgene.2022.1066118)

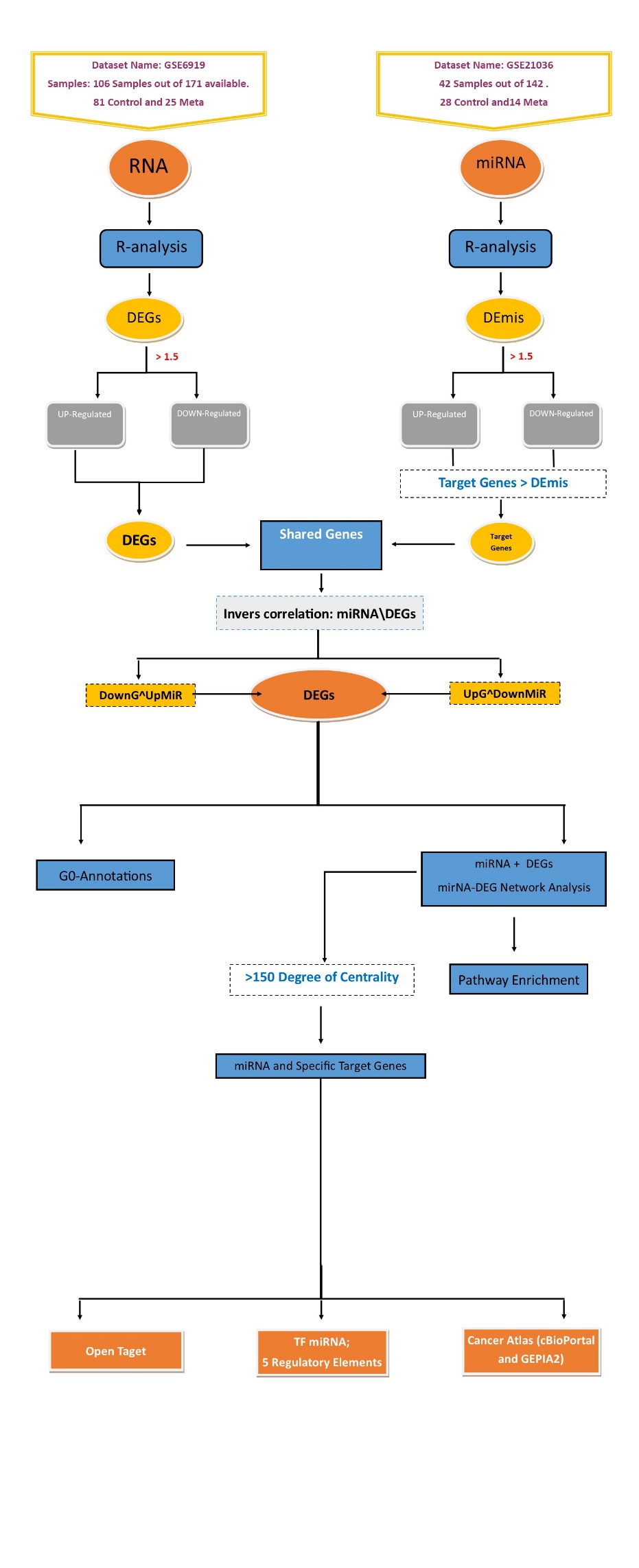
Supplementary Figure 1: Overall workflow of Current study

Supplement: Supplementary file 1 [file DataSheet2.docx]
