# Supplementary material for: A comparative mRNA- and miRNA transcriptomics reveals novel molecular signatures associated with metastatic prostate cancers
Source: Front Genet. 2022 Nov 16;13:1066118. doi: 10.3389/fgene.2022.1066118 (PMC9708707; doi:10.3389/fgene.2022.1066118)
Supplement: Supplementary file 2 [file DataSheet1.docx]

**Supplementary Table S1: The top 10 pathways enriched with miRNA-target genes in Prostate cancers**

| **Pathway** | **Total** | **Expected** | **Hits** | **P val.** |
| --- | --- | --- | --- | --- |
| Pathways in cancer | 310 | 135 | 212 | 6.28E-20 |
| HTLV-I infection | 199 | 86.7 | 138 | 5.45E-14 |
| Chronic myeloid leukaemia | 73 | 31.8 | 61 | 1.47E-12 |
| Prostate cancer | 87 | 37.9 | 69 | 6.49E-12 |
| Cell cycle | 124 | 54 | 91 | 8.57E-12 |
| Focal adhesion | 200 | 87.1 | 131 | 1.39E-10 |
| ErbB signalling pathway | 87 | 37.9 | 67 | 1.47E-10 |
| Glioma | 65 | 28.3 | 53 | 2.85E-10 |
| Neurotrophin signalling pathway | 123 | 53.6 | 86 | 1.95E-09 |
| Non-small cell lung cancer | 52 | 22.6 | 43 | 6.33E-09 |

**Supplementary Table S2: Open Target platform predictions showing drug name, ID, type, class, mechanism, disease name, clinical trial phase and its status of the three prostate cancer hub genes.**

| **Gene** | **Drug ID** | **Drug Name** | **Type of small molecule** | **Mechanism Of Action** | **Class** | **Disease Name** | **Clinical Trail Phase** | **Clinical Trail status** |
| --- | --- | --- | --- | --- | --- | --- | --- | --- |
| FGF2 | CHEMBL4297163 | MUPARFOSTAT | Oligosaccharide | Basic fibroblast growth factor inhibitor | Inhibitor | hepatocellular carcinoma | 3 | Terminated |
|  | CHEMBL4297163 | MUPARFOSTAT | Oligosaccharide | Basic fibroblast growth factor inhibitor | Inhibitor | prostate cancer | 2 | Completed |
|  | CHEMBL4297163 | MUPARFOSTAT | Oligosaccharide | Basic fibroblast growth factor inhibitor | Inhibitor | hepatocellular carcinoma | 2 | Completed |
|  | CHEMBL4297163 | MUPARFOSTAT | Oligosaccharide | Basic fibroblast growth factor inhibitor | Inhibitor | non-small cell lung carcinoma | 2 | Completed |
|  | CHEMBL4297163 | MUPARFOSTAT | Oligosaccharide | Basic fibroblast growth factor inhibitor | Inhibitor | lung cancer | 2 | Completed |
|  | CHEMBL4297163 | MUPARFOSTAT | Oligosaccharide | Basic fibroblast growth factor inhibitor | Inhibitor | cutaneous melanoma | 1 | Completed |
|  | CHEMBL4297163 | MUPARFOSTAT | Oligosaccharide | Basic fibroblast growth factor inhibitor | Inhibitor | melanoma | 1 | Completed |
| GSK3B | CHEMBL1200826 | LITHIUM CARBONATE | Small molecule | Glycogen synthase kinase-3 inhibitor | Inhibitor | osteosarcoma | 4 | Recruiting |
|  | CHEMBL1200826 | LITHIUM CARBONATE | Small molecule | Glycogen synthase kinase-3 inhibitor | Inhibitor | differentiated thyroid carcinoma | 2 | Completed |
|  | CHEMBL1200826 | LITHIUM CARBONATE | Small molecule | Glycogen synthase kinase-3 inhibitor | Inhibitor | medullary thyroid gland carcinoma | 2 | Terminated |
|  | CHEMBL362558 | LY-2090314 | Small molecule | Glycogen synthase kinase-3 beta inhibitor | Inhibitor | leukemia | 2 | Completed |
|  | CHEMBL1200826 | LITHIUM CARBONATE | Small molecule | Glycogen synthase kinase-3 inhibitor | Inhibitor | brain cancer | 2 | Terminated |
|  | CHEMBL1200826 | LITHIUM CARBONATE | Small molecule | Glycogen synthase kinase-3 inhibitor | Inhibitor | neuroendocrine neoplasm | 2 | Completed |
|  | CHEMBL483465 | 9-ING-41 | Small molecule | Glycogen synthase kinase-3 beta inhibitor | Inhibitor | malignant glioma | 1 | Recruiting |
|  | CHEMBL1200826 | LITHIUM CARBONATE | Small molecule | Glycogen synthase kinase-3 inhibitor | Inhibitor | small cell lung carcinoma | 1 | Completed |
|  | CHEMBL483465 | 9-ING-41 | Small molecule | Glycogen synthase kinase-3 beta inhibitor | Inhibitor | Malignant Bone Neoplasm | 1 | Recruiting |
|  | CHEMBL1200826 | LITHIUM CARBONATE | Small molecule | Glycogen synthase kinase-3 inhibitor | Inhibitor | prostate cancer | 1 | Completed |
|  | CHEMBL362558 | LY-2090314 | Small molecule | Glycogen synthase kinase-3 beta inhibitor | Inhibitor | pancreatic carcinoma | 1 | Terminated |
|  | CHEMBL483465 | 9-ING-41 | Small molecule | Glycogen synthase kinase-3 beta inhibitor | Inhibitor | breast neoplasm | 1 | Recruiting |
|  | CHEMBL483465 | 9-ING-41 | Small molecule | Glycogen synthase kinase-3 beta inhibitor | Inhibitor | kidney cancer | 1 | Recruiting |
|  | CHEMBL362558 | LY-2090314 | Small molecule | Glycogen synthase kinase-3 beta inhibitor | Inhibitor | cancer | 1 | Completed |
|  | CHEMBL483465 | 9-ING-41 | Small molecule | Glycogen synthase kinase-3 beta inhibitor | Inhibitor | lung neoplasm | 1 | Recruiting |
|  | CHEMBL483465 | 9-ING-41 | Small molecule | Glycogen synthase kinase-3 beta inhibitor | Inhibitor | pancreatic adenocarcinoma | 1 | Recruiting |
|  | CHEMBL483465 | 9-ING-41 | Small molecule | Glycogen synthase kinase-3 beta inhibitor | Inhibitor | sarcoma | 1 | Recruiting |
|  | CHEMBL1200826 | LITHIUM CARBONATE | Small molecule | Glycogen synthase kinase-3 inhibitor | Inhibitor | Central Nervous System Neoplasm | 1 | Terminated |
|  | CHEMBL483465 | 9-ING-41 | Small molecule | Glycogen synthase kinase-3 beta inhibitor | Inhibitor | metastasis | 1 | Recruiting |
| NR3C1 | CHEMBL384467 | DEXAMETHASONE | Small molecule | Glucocorticoid receptor agonist | Agonist | prostate cancer | 4 | Completed |
|  | CHEMBL131 | PREDNISOLONE | Small molecule | Glucocorticoid receptor agonist | Agonist | prostate cancer | 4 | Completed |
|  | CHEMBL635 | PREDNISONE | Small molecule | Glucocorticoid receptor agonist | Agonist | prostate cancer | 4 | Completed |
|  | CHEMBL384467 | DEXAMETHASONE | Small molecule | Glucocorticoid receptor agonist | Agonist | metastatic prostate cancer | 4 | Recruiting |
|  | CHEMBL635 | PREDNISONE | Small molecule | Glucocorticoid receptor agonist | Agonist | metastatic prostate cancer | 4 | Recruiting |
|  | CHEMBL635 | PREDNISONE | Small molecule | Glucocorticoid receptor agonist | Agonist | prostate cancer | 4 | Active, not recruiting |
|  | CHEMBL635 | PREDNISONE | Small molecule | Glucocorticoid receptor agonist | Agonist | prostate cancer | 3 | Active, not recruiting |
|  | CHEMBL389621 | HYDROCORTISONE | Small molecule | Glucocorticoid receptor agonist | Agonist | prostate adenocarcinoma | 3 | Terminated |
|  | CHEMBL635 | PREDNISONE | Small molecule | Glucocorticoid receptor agonist | Agonist | prostate cancer | 3 | Not yet recruiting |
|  | CHEMBL635 | PREDNISONE | Small molecule | Glucocorticoid receptor agonist | Agonist | prostate cancer | 3 | Completed |
|  | CHEMBL384467 | DEXAMETHASONE | Small molecule | Glucocorticoid receptor agonist | Agonist | prostate adenocarcinoma | 3 | Active, not recruiting |
|  | CHEMBL635 | PREDNISONE | Small molecule | Glucocorticoid receptor agonist | Agonist | prostate carcinoma | 3 | Active, not recruiting |
|  | CHEMBL131 | PREDNISOLONE | Small molecule | Glucocorticoid receptor agonist | Agonist | prostate cancer | 3 | Completed |
|  | CHEMBL139835 | CYPROTERONE ACETATE | Small molecule | Glucocorticoid receptor antagonist | Antagonist | prostate cancer | 3 | Completed |
|  | CHEMBL635 | PREDNISONE | Small molecule | Glucocorticoid receptor agonist | Agonist | prostate carcinoma | 3 | Completed |
|  | CHEMBL131 | PREDNISOLONE | Small molecule | Glucocorticoid receptor agonist | Agonist | prostate adenocarcinoma | 3 | Active, not recruiting |
|  | CHEMBL131 | PREDNISOLONE | Small molecule | Glucocorticoid receptor agonist | Agonist | prostate carcinoma | 3 | Completed |
|  | CHEMBL650 | METHYLPREDNISOLONE | Small molecule | Glucocorticoid receptor agonist | Agonist | prostate cancer | 3 | Completed |
|  | CHEMBL131 | PREDNISOLONE | Small molecule | Glucocorticoid receptor agonist | Agonist | prostate cancer | 3 | Active, not recruiting |
|  | CHEMBL384467 | DEXAMETHASONE | Small molecule | Glucocorticoid receptor agonist | Agonist | prostate cancer | 3 | Terminated |
|  | CHEMBL389621 | HYDROCORTISONE | Small molecule | Glucocorticoid receptor agonist | Agonist | prostate cancer | 3 | Completed |
|  | CHEMBL635 | PREDNISONE | Small molecule | Glucocorticoid receptor agonist | Agonist | prostate adenocarcinoma | 3 | Recruiting |
|  | CHEMBL384467 | DEXAMETHASONE | Small molecule | Glucocorticoid receptor agonist | Agonist | prostate adenocarcinoma | 3 | Recruiting |
|  | CHEMBL650 | METHYLPREDNISOLONE | Small molecule | Glucocorticoid receptor agonist | Agonist | prostate cancer | 3 | Terminated |
|  | CHEMBL635 | PREDNISONE | Small molecule | Glucocorticoid receptor agonist | Agonist | prostate cancer | 3 | Recruiting |
|  | CHEMBL635 | PREDNISONE | Small molecule | Glucocorticoid receptor agonist | Agonist | prostate cancer | 3 | Terminated |
|  | CHEMBL635 | PREDNISONE | Small molecule | Glucocorticoid receptor agonist | Agonist | metastatic prostate cancer | 3 | Active, not recruiting |
|  | CHEMBL139835 | CYPROTERONE ACETATE | Small molecule | Glucocorticoid receptor antagonist | Antagonist | prostate cancer | 3 | Unknown status |
|  | CHEMBL139835 | CYPROTERONE ACETATE | Small molecule | Glucocorticoid receptor antagonist | Antagonist | prostate adenocarcinoma | 3 | Completed |
|  | CHEMBL131 | PREDNISOLONE | Small molecule | Glucocorticoid receptor agonist | Agonist | prostate adenocarcinoma | 3 | Completed |
|  | CHEMBL635 | PREDNISONE | Small molecule | Glucocorticoid receptor agonist | Agonist | prostate adenocarcinoma | 3 | Completed |
|  | CHEMBL384467 | DEXAMETHASONE | Small molecule | Glucocorticoid receptor agonist | Agonist | prostate cancer | 3 | Completed |
|  | CHEMBL131 | PREDNISOLONE | Small molecule | Glucocorticoid receptor agonist | Agonist | metastatic prostate cancer | 3 | Active, not recruiting |
|  | CHEMBL384467 | DEXAMETHASONE | Small molecule | Glucocorticoid receptor agonist | Agonist | prostate cancer | 3 | Active, not recruiting |
|  | CHEMBL635 | PREDNISONE | Small molecule | Glucocorticoid receptor agonist | Agonist | prostate adenocarcinoma | 3 | Active, not recruiting |
|  | CHEMBL635 | PREDNISONE | Small molecule | Glucocorticoid receptor agonist | Agonist | metastatic prostate cancer | 3 | Recruiting |
|  | CHEMBL635 | PREDNISONE | Small molecule | Glucocorticoid receptor agonist | Agonist | prostate adenocarcinoma | 3 | Terminated |
|  | CHEMBL384467 | DEXAMETHASONE | Small molecule | Glucocorticoid receptor agonist | Agonist | prostate cancer | 2 | Recruiting |
|  | CHEMBL635 | PREDNISONE | Small molecule | Glucocorticoid receptor agonist | Agonist | Prostate Small Cell Carcinoma | 2 | Recruiting |
|  | CHEMBL650 | METHYLPREDNISOLONE | Small molecule | Glucocorticoid receptor agonist | Agonist | metastatic prostate cancer | 2 | Recruiting |

**Supplementary Table S3: Mutations in the nine hub genes**

| Gene name | Protein Change | Domain | Mutation Type | Copy# | COSMIC | Allele Freq (T) | # Mut in Sample |
| --- | --- | --- | --- | --- | --- | --- | --- |
| ABHD2 | P283L | Abhydrolase_1: alpha/beta hydrolase fold (166 - 388) | Missense | Diploid | 1 | 0.03 | 408 |
|  | Y334C |  | Missense | Diploid | 2 | 0.21 | 71 |
|  | C326Y |  | Missense | Diploid |  | 0.41 | 6535 |
|  | A261V |  | Missense | Diploid |  | 0.45 | 26 |
|  | A12P |  | Missense | Diploid | 1 | 0.35 | 38 |
|  | Y334C |  | Missense | Diploid | 2 | 0.21 | 53 |
|  | V231I | Abhydrolase_1: alpha/beta hydrolase fold (166 - 388) | Missense |  |  | 0.60 | 68 |
|  | Y334C |  | Missense | Diploid | 2 | 0.21 | 46 |
|  | C326Y |  | Missense | Diploid |  | 0.42 | 6251 |
|  | Y334C |  | Missense |  | 2 | 0.21 | 57 |
|  | C326Y |  | Missense |  |  | 0.41 | 6042 |
|  | V231I |  | Missense |  |  | 0.60 | 130 |
|  | M264V | Abhydrolase_1: alpha/beta hydrolase fold (166 - 388) | Missense |  |  | 0.37 | 1281 |
| FGF2 | *289Wext*2 |  | Nonstop | Amp |  | 0.03 | 122 |
|  | *289Wext*2 |  | Nonstop | Gain |  | 0.03 | 131 |
| DCAF7 | W24L |  | Missense | Gain | 2 | 0.0070 | 163 |
| GSK3B | R167* | Pkinase: Protein kinase domain (56 - 340) | Nonsense |  | 1 |  | 16 |
|  | R167* |  | Nonsense | ShallowDel | 1 | 0.31 | 32 |
|  | D31Y |  | Missense |  |  |  | 29 |
|  | A83T | Pkinase: Protein kinase domain (56 - 340) | Missense | Diploid | 2 | 0.35 | 5 |
|  | R180Q |  | Missense | Diploid | 3 | 0.23 | 78 |
|  | R220Q |  | Missense | Diploid |  | 0.15 | 33 |
|  | R148Q | Pkinase: Protein kinase domain (56 - 340) | Missense | Diploid |  | 0.36 | 29 |
|  | A143G | Pkinase: Protein kinase domain (56 - 340) | Missense | Diploid |  | 0.06 | 5 |
|  | R113H | Pkinase: Protein kinase domain (56 - 340) | Missense | Diploid |  | 0.46 | 45 |
|  | T277Nfs*16 |  | FS ins | Diploid |  | 0.23 | 2 |
|  | T235_S236del |  | IF del | Diploid |  | 0.4 | 4 |
|  | X301_splice | Pkinase: Protein kinase domain (56 - 340) | Splice | Diploid | 1 | 0.31 | 5 |
|  | A42T |  | Missense | Diploid |  | 0.11 | 192 |
|  | R321* |  | Nonsense | Diploid |  | 0.58 | 59 |
|  | T39S |  | Missense | Diploid |  | 0.2 | 539 |
|  | S351* |  | Nonsense | Diploid |  | 0.56 | 4 |
|  | D354* |  | FS ins | Diploid |  | 0.13 | 88 |
|  | R180Q |  | Missense |  | 3 |  | 710 |
|  | D31Y |  | Missense |  |  |  | 21 |
|  | T405R |  | Missense |  |  | 0.38 | 68 |
|  | R148Q |  | Missense | Diploid |  |  | 29 |
|  | A143G |  | Missense | Diploid |  |  | 5 |
|  | R167* |  | Nonsense | ShallowDel | 1 | 0.32 | 26 |
|  | E211Dfs*17 | Pkinase: Protein kinase domain (56 - 340) | FS del | Diploid |  | 0.17 | 19 |
|  | R220Q |  | Missense | Diploid |  | 0.15 | 33 |
|  | T235_S236del |  | IF del | Diploid |  | 0.4 | 4 |
|  | R321* |  | Nonsense | Diploid |  | 0.58 | 59 |
|  | R167* |  | Nonsense | Diploid | 1 | 0.31 | 24 |
|  | E211Dfs*17 |  | FS del | Diploid |  | 0.17 | 26 |
|  | T405R |  | Missense | Diploid |  | 0.38 | 130 |
|  | A143G |  | Missense |  |  | 0.06 | 5 |
|  | T277Nfs*16 | Pkinase: Protein kinase domain (56 - 340) | FS ins |  |  | 0.23 | 2 |
|  | T235_S236del |  | IF del |  |  | 0.4 | 4 |
|  | FSTL1-GSK3B fusion |  | Fusion | Diploid |  |  | 4 |
|  | FSTL1-GSK3B fusion |  | Fusion | Diploid |  |  | 4 |
|  | GSK3B-LSAMP |  | Fusion | ShallowDel |  |  | 106 |
|  | GSK3B-ARNT |  | Fusion | Diploid |  |  | 37 |
|  | GSK3B-COX17 |  | Fusion | DeepDel |  |  | 26 |
|  | GSK3B-TSC22D2 |  | Fusion | Gain |  |  | 26 |
|  | GSK3B-ATP11B |  | Fusion | Diploid |  |  | 29 |
|  | GSK3B-CD80 |  | Fusion | Gain |  |  | 22 |
|  | GSK3B-PLA1A |  | Fusion | Diploid |  |  | 40 |
|  | GSK3B-SEMA5B |  | Fusion | Gain |  |  | 55 |
|  | FSTL1-GSK3B fusion |  | Fusion |  |  |  | 4 |
| NACC2 | A544P |  | Missense | Diploid |  | 0.15 | 132 |
| NFIB | S246R | CTF_NFI: CTF/NF-I family transcription modulation region (209 - 415) | Missense | Diploid |  | 0.18 | 86 |
|  | P313L | CTF_NFI: CTF/NF-I family transcription modulation region (209 - 415) | Missense | ShallowDel |  | 0.05 | 58 |
|  | H14N | NfI_DNAbd_pre-N: Nuclear factor I protein pre-N-terminus (5 - 47) | Missense | Diploid |  | 0.05 | 148 |
|  | X309_splice | CTF_NFI: CTF/NF-I family transcription modulation region (209 - 415) | Splice | Diploid |  | 0.4 | 6535 |
|  | P313L |  | Missense | ShallowDel |  | 0.05 | 77 |
|  | X309_splice |  | Splice | Diploid |  | 0.39 | 6251 |
|  | P313L |  | Missense | ShallowDel |  | 0.05 | 61 |
|  | X309_splice |  | Splice | Diploid |  | 0.4 | 6042 |
|  | S292Lfs*25 | CTF_NFI: CTF/NF-I family transcription modulation region (209 - 415) | FS del | Diploid |  | 0.19 | 19 |
|  | H14N | NfI_DNAbd_pre-N: Nuclear factor I protein pre-N-terminus (5 - 47) | Missense | Diploid |  | 0.04 | 148 |
| DICER1 | C1354* | Ribonuclease_3: Ribonuclease III domain (1313 - 1575) | Nonsense | Diploid |  | 0.03 | 122 |
|  | S1076Wfs*12 |  | FS del | Diploid |  | 0.38 | 6 |
|  | K1362Rfs*17 | Ribonuclease_3: Ribonuclease III domain (1313 - 1575) | FS del | Diploid |  | 0.35 | 1081 |
|  | C1354* |  | Nonsense | Diploid |  | 0.03 | 131 |
|  | P737Qfs*21 |  | FS del | Diploid |  | 0.16 | 10 |
|  | K1362Rfs*17 |  | FS del |  |  | 0.35 | 822 |
|  | C1354* |  | Nonsense |  |  | 0.03 | 117 |
|  | X103_splice | DEAD: DEAD/DEAH box helicase (46 - 208) | Splice |  |  | 0.27 | 107 |
|  | S1076Wfs*12 |  | FS del |  |  | 0.38 | 6 |
|  | DICER1-intragenic |  | Fusion | Diploid |  |  | 3 |
|  | T566I |  | Missense | Diploid |  | 0.03 | 132 |
|  | Q140R | DEAD: DEAD/DEAH box helicase (46 - 208) | Missense | Diploid |  | 0.05 | 950 |
|  | T96M |  | Missense | Diploid |  | 0.02 | 84 |
|  | D1699N |  | Missense | ShallowDel |  | 0.11 | 36 |
|  | E1167Q |  | Missense | Diploid |  | 0.19 | 2 |
|  | R944Q | PAZ: PAZ domain (894 - 1064) | Missense | Diploid | 2 | 0.3 | 208 |
|  | V680A | Dicer_dimer: Dicer dimerisation domain (630 - 721) | Missense | Diploid | 1 | 0.12 | 51 |
|  | R1060H | PAZ: PAZ domain (894 - 1064) | Missense | Diploid | 2 | 0.09 | 28 |
|  | G1295V |  | Missense | Diploid |  | 0.31 | 4 |
|  | E382K |  | Missense | Diploid | 2 | 0.07 | 12 |
|  | Y1242H |  | Missense | Diploid |  | 0.43 | 1 |
|  | E970L | PAZ: PAZ domain (894 - 1064) | Missense | Diploid |  | 0.08 | 4 |
|  | K833M |  | Missense | Diploid |  | 0.07 | 6 |
|  | P365S |  | Missense | Diploid |  | 0.55 | 4 |
|  | K1516R | Ribonuclease_3: Ribonuclease III domain (1313 - 1575) | Missense | Diploid | 1 | 0.12 | 76 |
|  | S1543F |  | Missense | Diploid |  | 0.12 | 192 |
|  | S1637L |  | Missense | Diploid |  | 0.11 | 87 |
|  | E1420dup | Ribonuclease_3: Ribonuclease III domain (1313 - 1575) | IF ins | Diploid |  | 0.17 | 87 |
|  | N1668T |  | Missense | Diploid |  | 0.08 | 5 |
|  | I557F |  | Missense | Diploid | 1 | 0.31 | 3 |
|  | A1306T |  | Missense | Diploid |  | 0.13 | 88 |
|  | S1632F |  | Missense | Diploid |  | 0.14 | 20 |
|  | P674H | Dicer_dimer: Dicer dimerisation domain (630 - 721) | Missense | Diploid | 1 | 0.16 | 73 |
|  | P1869A |  | Missense | Diploid |  | 0.1 | 124 |
|  | R1703C | Ribonuclease_3: Ribonuclease III domain (1702 - 1824) | Missense | Diploid | 3 |  | 710 |
|  | T566I |  | Missense | Diploid |  | 0.03 | 164 |
|  | Q140R |  | Missense |  |  | 0.05 | 754 |
|  | E382K |  | Missense | Diploid | 2 |  | 12 |
|  | Y1242H |  | Missense | Diploid |  |  | 1 |
|  | L616S |  | Missense | Diploid |  |  | 40 |
|  | L616S |  | Missense | Diploid |  |  | 36 |
|  | D1699N |  | Missense | ShallowDel |  | 0.1 | 26 |
|  | N1921S |  | Missense |  |  |  | 13 |
|  | E382K |  | Missense | Diploid | 2 | 0.07 | 12 |
|  | Y1242H |  | Missense | Diploid |  | 0.43 | 1 |
|  | K833M |  | Missense | Diploid |  | 0.07 | 6 |
|  | K1516R |  | Missense | Diploid | 1 | 0.12 | 76 |
|  | E503K | Helicase_C: Helicase conserved C-terminal domain (495 - 554) | Missense | Diploid |  | 0.06 | 6 |
|  | D1699N |  | Missense |  |  | 0.11 | 24 |
|  | T566I |  | Missense |  |  | 0.03 | 114 |
|  | Q140R |  | Missense |  |  | 0.05 | 931 |
|  | A518V | Helicase_C: Helicase conserved C-terminal domain (495 - 554) | Missense |  |  | 0.25 | 27 |
|  | G1295V |  | Missense |  |  | 0.31 | 4 |
|  | E382K |  | Missense |  | 2 | 0.07 | 12 |
|  | Y1242H |  | Missense |  |  | 0.43 | 1 |
|  | E970L |  | Missense |  |  | 0.08 | 4 |
|  | K833M |  | Missense |  |  | 0.07 | 6 |
|  | P365S |  | Missense |  |  | 0.55 | 4 |
| NR3C1 | X632_splice | Hormone_recep: Ligand-binding domain of nuclear hormone receptor (544 - 735) | Splice | Diploid |  | 0.21 | 50 |
|  | X632_splice |  | Splice | Diploid |  | 0.21 | 47 |
| FGFR1OP (CEP43) | D70= | FOP_dimer: FOP N terminal dimerisation domain (54 - 134) | Splice | Diploid |  | 0.22 | 6535 |
|  | D70= |  | Splice | Diploid |  | 0.23 | 6251 |
|  | D70= |  | Splice | Diploid |  | 0.22 | 6042 |

**Supplementary Table S4: Localization of the expression of the nine hub genes in cancer cell lines from ATLAS**

| **Gene** | **Antibody** | **Cell Line** | **nTPM** | **Location** |
| --- | --- | --- | --- | --- |
| ABHD2 | HPA005999 | U-251 MG | 55.8 | Cytosol |
| DCAF7 | HPA022948 | U-251 MG | 54.4 | Nucleoplasm |
| FGF2 | CAB000125 | U-251 MG | 20.0 | Nucleoplasm & Nuclear bodies |
| GSK3B | HPA028017 | A-431 | 59.3 | Nucleoplasm |
| NACC2 | HPA052962 | HeLa | 33.3 | Nucleoli & Mitochondria |
| NFIB | HPA003956 | A-431 | 19.7 | Nucleoplasm & Nucleoli fibrillar center |
| DICER1 | CAB068185 | A-431 | 18.6 | Cytosol |
| NR3C1 | CAB010435\ HPA004248 | U-251 MG | 35.9 | Nucleoplasm & Cytosol |
| FGFR1OP | HPA071876 | Hep G2 | 58.2 | Centrosome |

Footnote: U-251 MG- glioblastoma, A-431- breast cancer, HeLa- cervical cancer, Hep G2- hepatic cancer. nTPM- normalized transcription per million
